# No Effect of Vitamin C Administration on Neutrophil Recovery in Autologous Stem Cell Transplantation for Myeloma or Lymphoma: A Blinded, Randomized Placebo-Controlled Trial

**DOI:** 10.3390/nu14224784

**Published:** 2022-11-11

**Authors:** Gwendolyn N. Y. van Gorkom, Lara S. Boerenkamp, Birgit L. M. G. Gijsbers, Heidi H. van Ojik, Will K. W. H. Wodzig, Lotte Wieten, Catharina H. M. J. Van Elssen, Gerard M. J. Bos

**Affiliations:** 1 Department of Internal Medicine, Division of Hematology, GROW School for Oncology and Developmental Biology, Maastricht University Medical Center, 6229 HX Maastricht, The Netherlands; 2 Central Diagnostic Laboratory, Department of Clinical Chemistry, Maastricht University Medical Center, 6229 HX Maastricht, The Netherlands; 3 Department of Transplantation Immunology, Maastricht University Medical Center, 6229 HX Maastricht, The Netherlands

**Keywords:** vitamin C, autologous hematopoietic stem cell transplantation, neutrophil

## Abstract

Vitamin C is an important micronutrient for various immune cells. It increases phagocytic cell function and is necessary for T and natural killer (NK) cell development. Patients in need of an autologous hematopoietic stem cell transplantation (HSCT) are often vitamin C-depleted. We therefore hypothesized that vitamin C supplementation could improve immune recovery in autologous HSCT patients. This blinded, placebo-controlled trial included 44 patients randomized to receive vitamin C or a placebo. The following outcome measures used were clinical and immunological parameters, among others: time to neutrophil recovery, serum, and intracellular vitamin C values. Twenty-one patients received vitamin C, and 23 received a placebo. The time to neutrophil recovery did not differ between the two groups at 11.2 days (*p* = 0.96). There were no differences in hospitalization time (19.7 vs. 19.1 days, *p* = 0.80), the incidence of neutropenic fever (57% vs. 78%, *p* = 0.20), or 3-month overall survival (90.5% vs. 100%, *p* = 0.13). Bacteremia seemed to occur less in the vitamin C group (10% vs. 35%, *p* = 0.07). Our study shows no benefit from vitamin C supplementation on neutrophil recovery and hospitalization, despite possible lower rates of bacteremia in the vitamin C group. Therefore, we do not advise vitamin C supplementation in this treatment group.

## 1. Introduction

For decades, vitamin C has been thought to be beneficial in treating cancer because of its function as an antioxidant. In the 1970s, Cameron and Pauling reported increased survival in advanced-stage cancer patients treated with high-dose intravenous vitamin C [1,2]. However, this effect could not be repeated by others, even though it was investigated in various clinical trials [3,4,5]. Vitamin C is important in phagocytic cells, such as neutrophils, where it accumulates and can enhance chemotaxis, phagocytosis, the generation of reactive oxygen species, and ultimately microbial killing [6]. It is also needed for apoptosis and the clearance of the spent neutrophils from sites of infection by macrophages [6,7,8]. In several studies, an association between vitamin C supplementation and granulocyte proliferation has been found [8,9,10]. In vitro, we observed another function of vitamin C that might be beneficial for the treatment of cancer. Namely, the need for vitamin C for T cell differentiation and vitamin C positively affects the proliferation of T cells and natural killer (NK) cells [11,12].

Cancer patients receiving chemotherapy that is toxic for stem cells and therefore undergoing hematopoietic stem cell transplantation (HSCT) have low immunity during a longer period of time. This is due to a period of neutropenia and an even longer period of lymphocytopenia [13]. The dietary intake of these patients is often diminished, and we showed significantly lower serum vitamin C levels in this group of patients [14]. As vitamin C is an important micronutrient for the immune system, perhaps the supplementation of vitamin C to patients during, and recovering, after stem cell transplantation could lead to faster immune reconstitution and therefore better clinical outcomes. Vitamin C supplementation is easy, cheap, and safe in healthy volunteers and in previous clinical studies on cancer patients [15,16].

In this randomized Phase-III study, we investigated the role of vitamin C supplementation on the immune recovery of patients that received high-dose chemotherapy in combination with an autologous stem cell transplantation (SCT) to rescue the bone marrow toxic effect of the high-dose treatment.

## 2. Materials and Methods

### 2.1. Observational Study

To investigate serum vitamin C concentrations of hematologic cancer patients, an observational study was performed at the hematology ward of the Maastricht University Medical Center (MUMC), Maastricht, The Netherlands. In this study, patients receiving an autologous stem cell transplantation for multiple myeloma, lymphoma, or acute leukemia were included. During the in-hospital period of the treatment, a weekly blood sample was taken and analyzed for serum vitamin C concentration. We compared these concentrations in time, and with vitamin C serum concentrations of their healthy family members who were visiting them were used as controls.

### 2.2. Run-In Phase

To determine the magnitude of the effect of intravenous vitamin C supplementation on serum vitamin C levels in autologous SCT patients, the intervention study was started with a run-in phase. The goal was to verify if the prescribed dose of vitamin C (70 mg per kilogram bodyweight per day) was sufficient (in accordance with reference values in healthy people).

To calculate sample size for this run-in period we used Simon’s two-stage design. In our observational reference group only 10% of patients had an optimal (>50 µM) vitamin C concentration in the serum on day 14. To see if the dosage of vitamin C was adequate, three patients were necessary. If all of the patients had a concentration of serum vitamin C of > 50 µM on day 14, we intended to continue with the randomized part of the trial with this dosage. If not, the run-in phase would be repeated with an increased dose until the optimal concentration at day 14 was shown in three patients.

### 2.3. Patient Enrolment of Randomized, Placebo-Controlled Phase

The study design was a single-center, prospective, triple-blind, randomized placebo-controlled trial. It investigated the benefit of vitamin C supplementation in patients undergoing an autologous stem cell transplantation for multiple myeloma or lymphoma at MUMC, Maastricht, The Netherlands. The study was approved by the medical ethical committee from the MUMC (NL68010.068.18) and registered at clinicaltrials.gov (NCT03964688). The study was performed in accordance with the provisions of the Declaration of Helsinki and Good Clinical Practice guidelines as defined by the International Conference on Harmonization. Patients scheduled to be admitted for autologous stem cell transplantation for multiple myeloma or lymphoma were asked to participate by their treating physician. Written informed consent was obtained from all patients before enrolment. Study inclusion followed if patients met all of the following criteria: 18 years or older, willing to give written informed consent, a diagnosis of multiple myeloma or lymphoma, in need of intensive chemotherapy plus autologous stem cell transplantation as standard care, and the presence of a central venous catheter. Key exclusion criteria included the following: inability to understand the nature and extent of the trial and the procedures required, history of kidney stones, estimated glomerular filtration rate (eGFR) less than 30 mL/min, glucose-6-phosphatedehydrogenase (G6PD) deficiency, life expectancy less than 1 month, the use of immunosuppressive medication other than chemotherapy and corticosteroids, and active AA supplementation other than normal daily multivitamin use.

The primary endpoint was the day of repopulation after the autologous stem cell transplantation. This was defined as the day the neutrophil count returned to at least 0.5 × 10^9^/L after a period of neutropenia. Sample size calculations were derived from the mean and standard deviation (SD) of neutrophil recovery of 216 patients between 2014 and 2016 in our center. This indicated that a total of 36 participants (18 per group) would have 90% power to detect a relevant effect (decrease of 1.5 days) with 5% type I error (R core team, 2020). Therefore, a total of 44 participants were enrolled to account for an anticipated 20% loss due to withdrawal.

### 2.4. Administration of Intervention

For the study, subjects were individually randomized with a 1:1 ratio between the intervention group (Arm A: vitamin C), and the control group (Arm B: placebo). Randomization was performed after the baseline measures were recorded and the in- and exclusion criteria were verified in an electronic web-based case report file. The randomization sequence was generated with Alea. Patients were stratified according to disease (myeloma or lymphoma). Treating clinicians, nursing staff, and participants were masked to group allocation, but unmasking could be requested by the treating clinician in case of emergencies. Outcome assessors were also masked for group allocation.

Patients in the active intervention group received intravenous vitamin C every 24 h in sodium chloride 0.9% in the dose of 70 mg per kilogram body weight starting on the first day of the intensive chemotherapy. This was administered in a 250 mL infusion bag over 3 h through a central venous line. Patients in the placebo group received 250 mL intravenous sodium chloride 0.9% delivered and administered the same as for the intervention group. The intravenous vitamin C and placebo were given daily until the day of discharge. After discharge patients in the intervention group received oral vitamin C twice a day in two capsules of 500 mg until day 42 of the study. The patients in the placebo group received the same number of identical-looking capsules filled with methylcellulose during the same period.

### 2.5. Collection of Clinical Data

Data were collected and managed using MACRO electronic data capture, a secure, web-based data collection, and storage tool. The data were de-identified using a patient study code. The following demographic and clinical data were collected at baseline: age, gender, hematological diagnosis, data of diagnosis, number and names of previous treatments, weight, length, ethnicity, performance status, prior autologous stem cell transplantation, and co-morbidities. In addition, the following clinical data were collected: incidence of neutropenic fever, days of fever (day with temperature 38.5 °C or higher), incidence of bacteremia, use of antibiotics, date of repopulation (the day the neutrophil count returned to at least 0.5 × 10^9^/L after a period of neutropenia), days of hospitalization, incidence of readmission, relapse within 3 months and survival after 3 months.

All adverse events grade 3 or higher, other than those considered as standard for intensive chemotherapy (alopecia, nausea, vomiting, diarrhea, and hematological toxicities), were recorded as per the *Common Terminology Criteria for Adverse Events* (CTCAE, version 5.0).

### 2.6. Collection and Analysis of Blood Samples

Blood samples were collected almost daily for routine hematological and biochemical parameters and were analyzed at the central diagnostic laboratory the MUMC, which has acquired ISO 15189 accreditation for these routine measurements.

Study-related blood samples were collected at the following: baseline, day 8 of the study, repopulation, and after the completion of the intervention (day 42). Five ml blood from a serum tube was used for the determination of the serum vitamin C level using a commercial kit from Chromsystems (Gräfelfing, Germany). The intracellular vitamin C concentration of peripheral blood mononuclear cells (PBMCs) was measured by HPLC with UV detection from 8 mL heparinized blood, as described previously (17). This was performed at baseline, repopulation, and at the end of the intervention period using a 4 mL EDTA blood sample for the absolute cell counts of lymphocyte subsets by flowcytometry with BD Trucount tubes (BD Biosciences, Franklin Lakes, NJ, USA) using BD Multitest immunofluorescence reagents (CD3 FITC/CD8 PE/CD45 PerCP/CD4 APC and CD3 FITC/CD16 + CD56 PE/CD45 PerCP/CD19 APC, BD Biosciences) measured on a BD FACSCanto or FACSLyric flow cytometry system (BD Biosciences).

### 2.7. Collection of Quality of Life Data

The concept of health-related quality of life (HRQoL) has gained much interest during the last decades and is considered a secondary endpoint for evaluation of HRQoL during and after cancer treatment. Several instruments measuring HRQoL are applied in the field of oncology, with the ‘European Organization for Research and Treatment of Cancer’ (EORTC) Quality of Life Core Questionnaire QLQ-C30 being the instrument most frequently used in clinical trials and is the standard in Europe. The QLQ-C30 is a 30-item instrument, has been extensively tested for reliability and validity, and is translated into Dutch. The QLQ-C30 addresses six multi-item scales of functioning (physical, role, social, emotional, and cognitive functioning, and global health). Furthermore, three multi-item scales (fatigue, nausea and vomiting, and pain) and six single items (dyspnea, constipation, sleeping problems, appetite loss, diarrhea, and financial problems) are related to symptoms or problems. We asked participants to fill in this questionnaire the following three times: at baseline, at discharge from the hospital, and at the end of the study period.

### 2.8. Statistical Analyses

The baseline characteristics of the study population were described by presenting categorical data as proportions and continuous variables as means (+standard deviation). For all continuous variables, we used an unpaired two-tailed t-test to investigate if there were differences between the two treatment groups. For all categorical variables, we used a Chi-squared test to investigate if there were significant differences between vitamin C and placebo treatment. A Pearson’s correlation coefficient was used to investigate the association between PBMC and serum vitamin C concentration. Time until event data were analyzed using Kaplan–Meier plots with a log-rank test.

For the majority of the QLQ-C30 items, a four-point Likert-type response scale is used. Exceptions are the items for the global quality-of-life scale (where a seven-point scale is used). A linear transformation to a “0–100” scale of the EORTC QLQ-C30 questionnaire was carried out according to the EORTC Scoring Manual. A higher mean score for global QoL and functional scales reflects a better level of functioning, but a higher mean score for symptoms reflects more problems. To compare immune cell and HRQoL data between the two treatment groups, a Mann–Whitney U test was used. To examine the difference in occurrence of adverse events between the two groups, a Fisher Exact test was used. All statistical analyses were performed using SPSS statistics for Windows (IBM Corp, Version 25.0. Armonk, NY, USA).

## 3. Results

### 3.1. Observational Study

**Figure 1:**
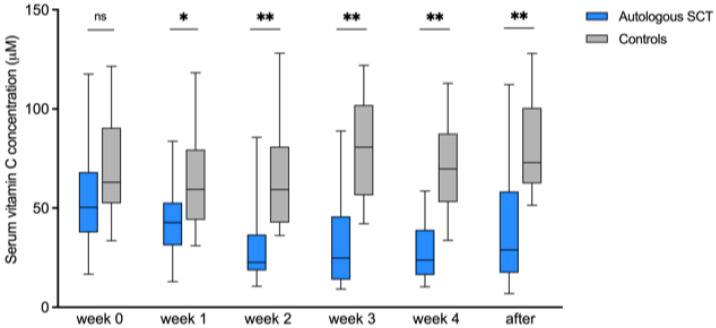
Vitamin C concentration in serum over time in autologous SCT patients vs. healthy controls. Vitamin C concentrations in µM in serum of patients receiving an autologous SCT, and of their healthy family members as controls. Week 0 is at baseline before the start of the myeloablative chemotherapy, after that blood samples were taken weekly. “After” is after discharge in the outpatient clinic on different time periods between 5 and 12 weeks after SCT. Box plots show median values with 25th and 75th percentiles as boundaries and whiskers indicate range, independent samples two-tailed T test was performed to compare the autologous SCT patients to the control group. SCT: stem cell transplantation. ns = not significant, * *p* < 0.05, ** *p* < 0.01.

The observational study was performed to investigate vitamin C levels in patients with various hematological malignancies before, during, and after admission to the hospital. We included 31 patients with autologous stem cell transplantation and 19 of their healthy family members as controls. The main hematological diagnosis was multiple myeloma (n = 20, 65%), followed by lymphoma (n = 7, 23%, and acute myeloid leukemia (n = 4, 13%). There was a slight male predominance (55%). The mean age of the patients was 57 years (range 20–67). At baseline, the serum vitamin C levels of the patients were similar to the controls. However, we discovered that more than 90% of the patients had a significant decrease in serum vitamin C levels during therapy. The serum vitamin C concentrations were also significantly lower than those of healthy family members followed over the same period from week 2 after the start of the chemotherapy, continuing even after discharge (Figure 1).

### 3.2. Run-In Phase

**Figure 2:**
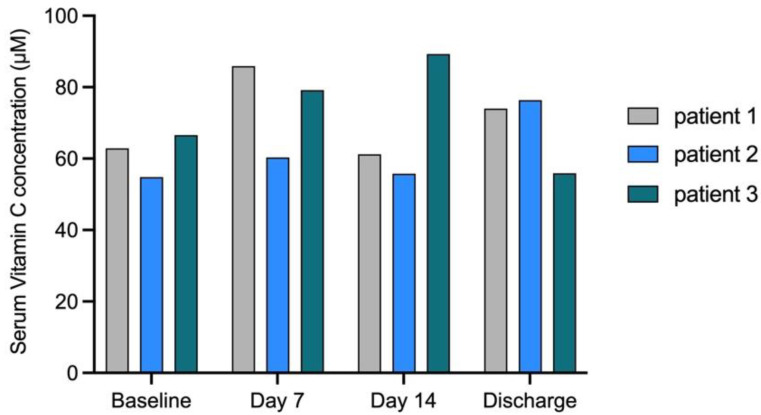
Vitamin C serum levels over time for the three patients of the run-in phase. Vitamin C serum levels over time for the first three patients in the run-in phase. Vitamin C supplementation of 70 mg/kg/day intravenously resulted in all patients in an optimal vitamin C concentration >50 µM not only at day 14 of the study, but at all of the time points.

We included three patients who also underwent high-dose chemotherapy and autologous SCT in the run-in phase of the study to investigate if the amount of vitamin C supplementation was adequate. The mean concentration of serum vitamin C at day 14 in the 3 patients of the run-in phase at the dosage of 70 mg per kilogram body weight per day was 69 µM, and all of the values were >50 µM, which is considered the optimal concentration (Figure 2) [17].

After the evaluation of the run-in phase, we started with the randomized placebo-controlled trial with this vitamin C dosage of 70 mg per kilogram of body weight per day, either intravenously or orally.

### 3.3. Randomized Placebo-Controlled Phase

#### 3.3.1. Patient Characteristics

**Figure 3:**
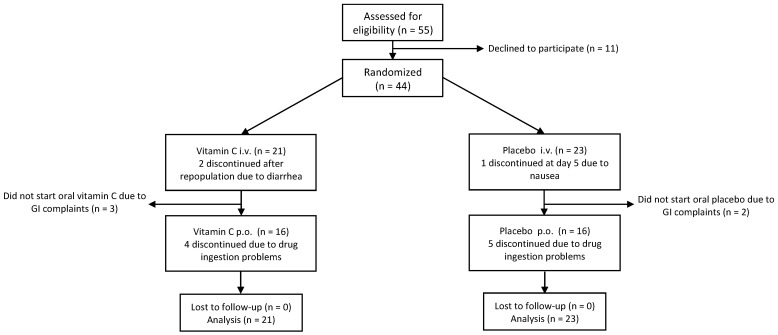
Flow diagram of randomized controlled phase.

Of the 55 patients assessed for eligibility, 44 were randomized to either the placebo or vitamin C arm (Figure 3). There was a slight female predominance, and the mean age was 61 years (Table 1). The most common diagnosis was multiple myeloma (68%). There were no significant differences between the two groups with respect to weight, length, body mass index (BMI), the presence of comorbidities, performance status, number of previous treatment lines, previous autologous SCT, and time since diagnosis.

#### 3.3.2. Vitamin C Kinetics

**Figure 4:**
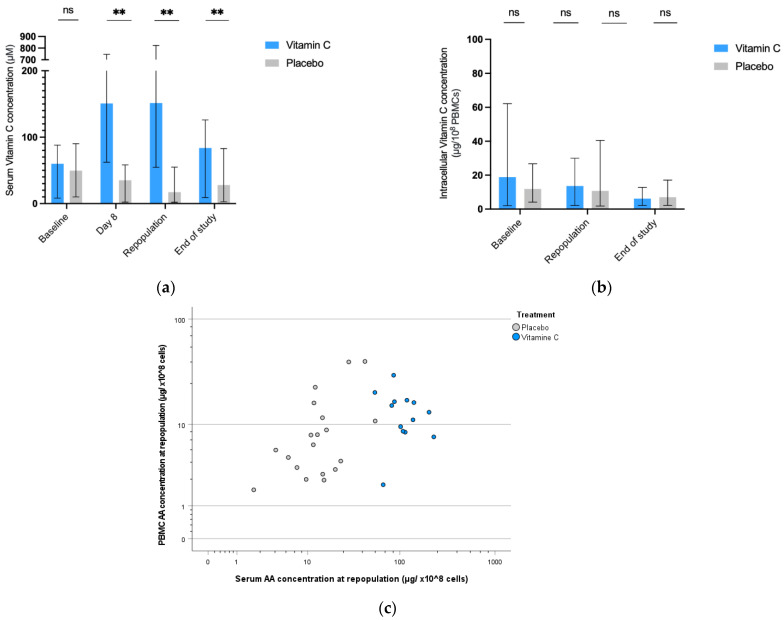
Vitamin C kinetics in serum and PBMCs. (**a**) Vitamin C kinetics in serum. Concentration (with range) of vitamin C in serum in µM determined in vitamin C and placebo group at baseline, day 8, repopulation and at the end of the study (around day 42). (**b**) Vitamin C kinetics in PBMCs. Concentration (with range) of vitamin C in PBMCs in µg/108 cells determined in vitamin C and placebo group at baseline, repopulation, and at the end of the study (around day 42). (**c**) Scatterplot of vitamin C in PBMCs versus serum levels at repopulation. Concentration of vitamin C in PBMC µg/108 cells versus serum in µM at repopulation in both groups on a logarithmic scale. Abbreviations: PBMCs: peripheral blood mononuclear cells. ns = non-significant, ** *p* < 0.005.

The vitamin C concentration in the serum at baseline was similar in the two treatment arms but was significantly higher in the vitamin C group at all other time points (day 8, repopulation and end of study) as was expected (Figure 4a). However, the mean intracellular vitamin C level/concentration in the PBMCs did not differ between the two treatment groups at any time (Figure 4b). Furthermore, there was no correlation between intracellular and serum vitamin C level at any moment (correlation coefficient (r^2^) 0–0.19)) in the vitamin C group. At repopulation there were less patients with low intracellular vitamin C concentrations in the vitamin C group. If the 10th percentile in healthy volunteers (14) is used as a cut-off (under 4.0 µg/10^8^ cells) the odds ratio (OR) is 5.5, *p* = 0.20), if the mean of healthy volunteer is used as cut-off (under 7.9 µg/10^8^ cells) OR is 7.6 (*p* = 0.03) (Figure 4c).

#### 3.3.3. Effect of Intervention on Neutrophil Engraftment

**Figure 5:**
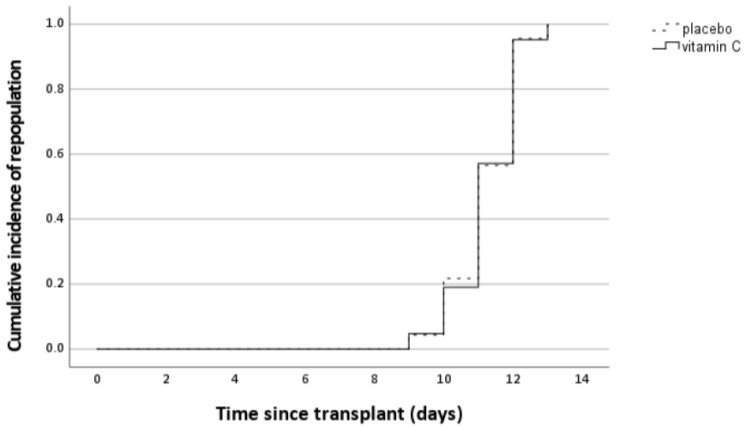
Kaplan–Meier plot for neutrophil recovery after the autologous stem cell transplantation. Lines indicate the percentage of patients that experience neutrophil recovery in days after stem cell transplantation. There was no significant difference between the intervention and the placebo group (*p* = 0.96, log rank test).

Neutrophil recovery was defined as the day the neutrophil reached a value of 0.5 × 10^9^ cells/L. The mean duration of neutrophil recovery after autologous SCT was 11.2 days (range 9–13) from the moment of transplantation. This was identical in both treatment groups (Figure 5).

#### 3.3.4. Effect of Intervention on Other Clinical Parameters

**Figure A1:**
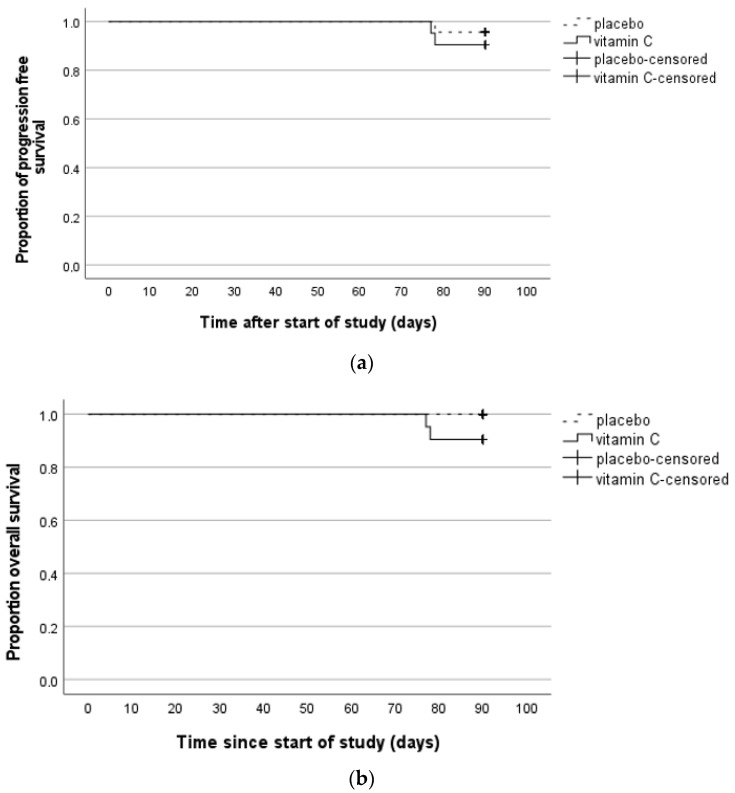
Progression-free and overall survival. (**a**) Kaplan–Meier plot of progression-free survival defined as time from the start of the study intervention to either relapse or death by treatment group. (**b**) Kaplan–Meier plot of overall survival defined as time from the start of the study intervention to death by treatment group.

The mean duration of hospital stay was 19.7 days in the intervention group versus 19.1 days in the placebo group, which was not significantly different (*p* = 0.80) (Table 2). The occurrence of neutropenic fever seemed less common in the intervention group (57% versus 78%); however, the study was underpowered to see any significant difference (*p* = 0.20). The duration of neutropenic fever was similar in both groups. However, the number of cases of bacteremia was higher in the placebo group than in the vitamin C group (35% versus 10%), but this did not lead to a significant difference in the use of antibiotic treatment. There were no relevant differences in the number of readmissions between the treatment groups. One patient in the placebo group relapsed within 3 months after the start of the study, and two of the patients in the vitamin C group died due to COVID-19 within 3 months. The overall survival and progression-free survival were not significantly different between the two groups (Figure A1a,b in Appendix A).

#### 3.3.5. Effect of Intervention on Quality of Life

**Figure 6:**
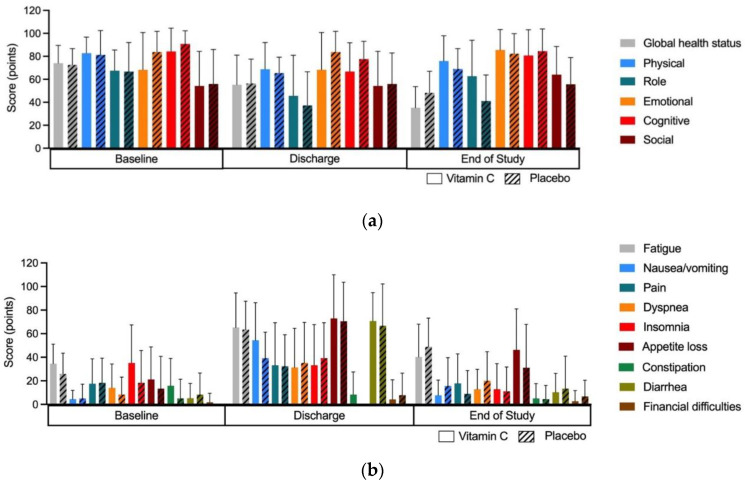
QLQ-EORTC-C30 outcomes. (**a**) Results of QLQ-EORTC-C30 global health status and function scales in vitamin C and placebo group at baseline, repopulation, and end of study. Bars represent mean and standard deviation. (**b**) Results of QLQ-EORTC-C30 symptom scales and single items in vitamin C and placebo group at baseline, repopulation, and end of study. Bars represent mean and standard deviation.

HRQoL was evaluated at baseline, at repopulation, and at the end of the study using the QLQ-EORTC-C30 quality of life questionnaire (Figure 6a,b). At baseline, the quality of life in general and symptom scales were comparable between the two treatment groups. At repopulation, the quality of life was generally lower, and symptoms occurred more often for both groups, as can be expected after high-dose chemotherapy. However, there were no significant differences between both groups. At the end of the study, the functional scale ‘role’ seemed better in the vitamin C group, meaning that they had fewer difficulties in performing their jobs and/or hobbies; however, this was not statistically significant (*p* = 0.10). The per-protocol analysis including only patients that completed the vitamin C or placebo treatment for all 6 weeks (vitamin C n = 12, placebo n = 15) also did not show statistical significance (*p* = 0.09).

#### 3.3.6. Effect of Intervention on Lymphocyte and NK Cell Immune Reconstitution

At baseline, the numbers of total lymphocytes, NK cells, B-, and T cells were comparable between the two treatment groups. However, at the time of repopulation, patients in the vitamin C group had significantly higher numbers of cytotoxic T cells. All other immune cells were comparable between the two treatment groups (Table 3). At the end of the study, the difference in cytotoxic T-cell levels was no longer present. Per-protocol analyses did not change any of these results.

#### 3.3.7. Adverse Events

Serious adverse events related to the intervention did not occur in the cohort. Grade 3 adverse events seemed more common in the treatment group; however, no statistical significance was shown (*p* = 0.76). Hypokalemia and mucositis were the most prevalent adverse events (Table 4). Other occurring adverse events included gastric hemorrhage, pneumonia, hypertension, toxicodermia, malnutrition, and hypomagnesemia. Two deaths occurred in the vitamin C group; both were COVID-19-related. No deaths occurred in the placebo group.

## 4. Discussion

This study aimed to investigate the effects of vitamin C supplementation in autologous SCT patients in the setting of a randomized controlled trial with a focus on immune recovery and clinical parameters.

In the observational study, we have shown that serum vitamin C levels of patients with various hematological disorders are comparable to those of healthy individuals before starting therapy, however, these levels decreased during therapy, and this effect remained present until after the therapy had ended. The run-in phase showed that intravenous vitamin C supplementation with 70 mg per kilogram of body weight per day increased the serum concentration to the desired value. These findings encouraged us to move forward with the randomized controlled trial.

In the clinical trial, we found that intravenous vitamin C supplementation had no effect on the time to neutrophil recovery in autologous stem cell transplantation patients. Several earlier studies describe an effect on the neutrophil function of vitamin C (chemotaxis, phagocytosis, microbial killing, and apoptosis); however, less is known about the role of vitamin C in neutrophil development. Despite the fact that there is a tendency toward a lower incidence of bacteriemia (10% in the vitamin group versus 35% in the placebo group (*p* = 0.07) there was no difference in the incidence of neutropenic fever or use of antibiotics, which is most likely associated with the function of granulocytes. Regardless of bacteremia, all patients with neutropenic fever receive antibiotic treatment for a minimal duration of 7 days or until the end of neutropenia; if any cultures come back positive, the antibiotic treatment will be adjusted accordingly. This standard protocol could partly explain why there is no difference seen in the type and duration of antibiotic treatment. Furthermore, there was no influence on the duration of hospitalization.

Vitamin C supplementation led to an increase in vitamin C in the serum compared to the placebo group. However, the increased serum concentration did not lead to a significant increase in the mean concentration of intracellular vitamin C in PBMCs. Moreover, there was hardly any correlation between the levels of vitamin C intracellularly and in the serum. The lack of a correlation between intracellular or tissue levels and plasma/serum vitamin C has also been shown in previous studies [15,18]. However, our data are in contrast with a study by Levine et al., who showed that the vitamin C concentration in immune cells could increase upon supplementation after a period of depletion [15]. At repopulation, there were fewer patients with a low concentration of intracellular vitamin C in the intervention group compared to the placebo group. We hypothesize that a high serum vitamin C level can prevent or correct low intracellular vitamin C levels but does not influence the intracellular level when it is already within a normal range.

Another possible explanation for the lack of effect on the mean intracellular vitamin C concentrations in our cohort could be that PBMCs serve as a vitamin C reservoir and most levels are not influenced by a short-lived period of vitamin C depletion in the serum, as is the case in these autologous SCT patients. However, it is clear that a larger group of patients do have low values (<10% of the reference level of the control group) while on a placebo compared to vitamin C treatment. This difference does disappear, however, at longer follow-up during the study.

A limitation of the study is that we were not able to measure intracellular levels in granulocytes for technical reasons. Nevertheless, T cell recovery seemed to be slightly faster in the vitamin C group, as they had higher numbers of cytotoxic T cells when compared with the placebo group at repopulation. This could be a realistic difference, as it is known that vitamin C is required for T cell development [11]. However, if we perform a Bonferroni correction for the multiple tests performed, this difference is no longer significant. Also, the T cell recovery advantage is not long-lasting for the vitamin C group, since at the end of the study, T cell numbers are comparable between the two treatment arms. Still, this finding asks for further investigation because faster T cell recovery or improved T cell function could lead to fewer viral infections. Even though we did not see a clinical effect in our patient group, it could be worthwhile to use vitamin C in other patient groups that show a longer period of temporary T cell depletion, for example, allogeneic SCT patients. These patients are known to have even lower vitamin C concentrations [14], therefore, supplementation could possibly be more effective in this group. If these results are translated to allogeneic SCT patients, there might also be some risk involved in vitamin C supplementation in this specific patient cohort. Faster T cell recovery could lead to more (early-onset) acute graft versus host disease (GVHD) [19]. Meanwhile, we have collected limited data on T cell phenotype and function, and therefore more research is needed before using vitamin C in this patient category. Currently, one study on this topic is registered at clinicaltrials.gov (NCT03613727), a phase II trial in which vitamin C is given to allogeneic SCT patients and non-relapse mortality is the primary endpoint [20].

The quality of life was comparable between the two groups. Intention-to-treat as well as per-protocol analysis for nearly significant categories showed no differences. Several studies on solid malignancy patients have shown that vitamin C supplementation can lead to the improvement of HRQoL and the reduction of symptoms [16,21,22,23]. However, most of these studies were of poor methodological quality, e.g., not placebo controlled. We were not able to reproduce this result in this hematologic malignancy patient cohort.

Adverse events occurred in both treatment arms with no statistical difference. Most occurring were hyperkaliemia and mucositis, two side effects that are frequently detected in patients treated with a high dose of melphalan. Diarrhea is very common in this patient group and, therefore, was not registered. Nevertheless, in the intervention group, two patients discontinued intravenous treatment because of diarrhea, and none in the placebo group. This could have been due to a synergistic effect between vitamin C and melphalan, as was seen in mice injected with a myeloma cell line. This synergy also created the possibility of decreasing the melphalan dose, while survival was not compromised in these mouse models [24]. Our study did not take this possible effect into account, and melphalan dosing was equal in both treatment arms. This set-up of the study could have led to more side effects of melphalan in the intervention arm. Perhaps it could also have led to deeper responses in the multiple myeloma patients, but as we did not gather minimal residual disease data, we have no results to present.

## 5. Conclusions

The results of this study do not show a significant or clinically relevant benefit of vitamin C supplementation in autologous SCT patients. It did not lead to quicker engraftment, nor did it lead to a shortened hospital stay. However, vitamin C supplementation might temporarily benefit T cell recovery and lead to diminished rates of bacteremia. The addition of vitamin C to the treatment regimens used seemed safe, as there were no serious adverse events and side effects were equal in the treatment arms. Taken together, though pre-clinical data show a beneficial effect of vitamin C on immune reconstitution and function, this study shows no clinical benefit of vitamin C supplementation in patients undergoing an autologous SCT. We, therefore, do not recommend vitamin C supplementation in this patient category.

## Figures and Tables

**Table 1:** Baseline characteristics of patients in randomized trial characteristics.

	Vitamin C, (n = 21)	Placebo, (n = 23)	*p* Value
Age (years), mean (range)	61.7 (31–71)	61.4 (34–72)	0.92
Gender			1.0
Male, n, (%)	9 (43)	10 (43)	
Female, n, (%)	12 (57)	13 (57)	
Disease			0.75
MM, n, (%)	15 (71)	15 (65)	
Lymphoma, n, (%)	6 (29)	8 (35)	
Weight (kg), mean (range)	72.7 (47–125)	75.7 (46–105)	0.60
Length (cm), mean (range)	169.1 (153–197)	169.3 (150–191)	0.96
BMI, mean (range)	25.1 (16.5–36.1)	26.2 (16.9–36)	0.43
Comorbidities, n, (%)			1.0
Yes:	12 (57)	13 (57)	
Cardiac	4	3	
Pulmonary	0	2	
Renal	1	1	
Hepatic	1	0	
Other	6	7	
No:	9 (43)	10 (43)	
WHO, n, (%)			0.57
0	13 (62)	15 (65)	
1	8 (38)	7 (30)	
2	0 (0)	1 (4)	
Number of previous treatment lines, n, (%)			0.62
1	14 (67)	15 (65)	
2	7 (33)	7 (30)	
>2	0 (0)	1 (4)	
Previous autologous transplantation, n, (%)			1.0
Yes	3 (14)	4 (17)	
No	18 (86)	19 (83)	
Time diagnosis to study (months), mean (range)	12.3 (4.0–77.7)	21.2 (4.1–137.1)	0.28

Abbreviations: MM; multiple myeloma, BMI: body mass index, WHO: World Health Organization.

**Table 2:** Effect of intervention on other clinical parameters.

	Vitamin C	Placebo	*p* Value
Duration of hospitalization (days), mean (range)	19.7 (13–47)	19.1 (14–31)	0.80
Neutropenic fever, n (%)			0.20
Yes:	12 (57)	18 (78)	
No:	9 (43)	5 (22)	
Duration of neutropenic fever (days), mean (range)	2.3 (1–4)	2.8 (1–7)	0.48
Duration of antibiotic treatment (days), mean (range)	3.7 (0–10)	4.8 (0–9)	0.28
Presence of bacteremia, n (%)			0.07
Yes:	2 (10)	8 (35)	
No:	19 (90)	15 (65)	
Readmission, n (%)			0.67
Yes:	2 (10)	4 (17)	
No:	19 (90)	19 (83)	
PFS 3 months	90.5% (SE 6.4)	95.7% (SE 4.3)	0.49
OS 3 months	90.5% (SE 6.4)	100%	0.13

Abbreviations: PFS: progression-free survival, OS: overall survival, SE: standard error.

**Table 3:** Effect of intervention on lymphocyte and NK cell immune reconstitution.

	**Baseline**			**Repopulation**			**End of Study**		
	**Vitamin C**	**Placebo**	***p***	**Vitamin C**	**Placebo**	***p***	**Vitamin C**	**Placebo**	***p***
Lymphocytes	1040.7 (497–2098)	1099.9 (352–221)	0.68	473.1 (10–1800)	289.5 (58–840)	0.10	1702.8 (415–3895)	1395.8 (469–3071)	0.35
NK cells	192.6 (4–863)	186.2 (76–373)	0.88	91.8 (3–715)	59.0 (1–220)	0.41	412.3 (130–1314)	293.2 (31–1005)	0.34
B cells	54.2 (0–148)	73.1 (0–288)	0.40	0.5 (0–4)	3.1 (0–47)	0.32	20.3 (0–130)	15.1 (0–126)	0.68
Total T cells	812.2 (309–1809)	833.4 (141–1872)	0.87	370.4 (6–1106)	216.8 (36–579)	0.06	1260.3 (162–3720)	1067.7 (350–2894)	0.57
Th cells	435.6 (76–952)	454.8 (28–745)	0.78	199.2 (5–618)	156.8 (31–431)	0.31	378.6 (98–1048)	318.0 (124–714)	0.47
Cytotoxic T cells	366.6 (47–909)	357.8 (73–1445)	0.92	173.4 (1–825)	60.8 (3–207)	0.03	842.9 (66–2638)	733.9 (46–2159)	0.69

Abbreviations: NK: natural killer, Th: T helper. All cell numbers are shown as: (cells/µL), mean (range).

**Table 4:** Adverse events.

	Vitamin C (n = 21)	Placebo (n = 23)	*p*-Value
Patients with AE (grade 3)	9	8	0.76
AE grade 3			
Hypokalemia	8	7	0.75
Mucositis	4	1	0.18
Gastric hemorrhage	0	1	1.0
Pneumonia	1	1	1.0
Hypertension	1	0	0.48
Toxicodermia	1	0	0.48
Malnutrition	1	0	0.48
Hypomagnesaemia	1	0	0.48
TOTAL	17	11	
Death	2 (COVID-19)	0	0.22

## Data Availability

The data presented in this study are available on reasonable request from the corresponding author.
